# Obesity-related health impacts of fuel excise taxation- an evidence review and cost-effectiveness study

**DOI:** 10.1186/s12889-017-4271-2

**Published:** 2017-05-04

**Authors:** V. Brown, M. Moodie, L. Cobiac, A. M. Mantilla Herrera, R. Carter

**Affiliations:** 1 Centre for Research Excellence in Obesity Policy and Food Systems, c/- Centre for Population Health Research, Faculty of Health, Deakin University, Geelong, VIC 3220 Australia; 2 Deakin Health Economics, Centre for Population Health Research, School of Health and Social Development, Deakin University, Geelong, VIC 3220 Australia; 3 Global Obesity Centre (GLOBE), World Health Organisation (WHO) Collaborating Centre for Obesity Prevention, School of Health and Social Development, Deakin University, Geelong, VIC 3220 Australia; 4 Centre for Health Policy, School of Population and Global Health, The University of Melbourne, Melbourne, Australia; 5 School of Public Health, The University of Queensland, Brisbane, Australia

**Keywords:** Active transport, Cost-effectiveness, Obesity, Physical activity

## Abstract

**Background:**

Reducing automobile dependence and improving rates of active transport may reduce the impact of obesogenic environments, thereby decreasing population prevalence of obesity and other diseases where physical inactivity is a risk factor. Increasing the relative cost of driving by an increase in fuel taxation may therefore be a promising public health intervention for obesity prevention.

**Methods:**

A scoping review of the evidence for obesity or physical activity effect of changes in fuel price or taxation was undertaken. Potential health benefits of an increase in fuel excise taxation in Australia were quantified using Markov modelling to simulate obesity, injury and physical activity related health impacts of a fuel excise taxation intervention for the 2010 Australian population. Health adjusted life years (HALYs) gained and healthcare cost savings from diseases averted were estimated. Incremental cost-effectiveness ratios (ICERs) were reported and results were tested through sensitivity analysis.

**Results:**

Limited evidence on the effect of policies such as fuel taxation on health-related behaviours currently exists. Only three studies were identified reporting associations between fuel price or taxation and obesity, whilst nine studies reported associations specifically with physical activity, walking or cycling. Estimates of the cross price elasticity of demand for public transport with respect to fuel price vary, with limited consensus within the literature on a probable range for the Australian context. Cost-effectiveness modelling of a AUD0.10 per litre increase in fuel excise taxation using a conservative estimate of cross price elasticity for public transport suggests that the intervention would be cost-effective from a limited societal perspective (237 HALYs gained, AUD2.6 M in healthcare cost savings), measured against a comparator of no additional increase in fuel excise. Under “best case” assumptions, the intervention would be more cost-effective (3181 HALYs gained, AUD34.2 M in healthcare cost savings).

**Conclusions:**

Exploratory analysis suggests that an intervention to increase fuel excise taxation may deliver obesity and physical activity related benefits. Whilst such an intervention has significant potential for cost-effectiveness, potential equity and acceptability impacts would need to be minimised. A better understanding of the effectiveness and cost-effectiveness of a range of transport interventions is required in order to achieve more physically active transport environments.

## Background

Physical inactivity is a global public health problem. Modern society has replaced many daily actions involving physical activity (PA) with motorised and computerised alternatives, and populations are now experiencing unprecedented levels of conditions such as obesity and other non-communicable diseases where physical inactivity is a risk factor. The increasing global burden of largely preventable diseases has led to recognition of the need for ‘upstream’ interventions for prevention [1, 2]. These interventions focus on macro-level factors and include government policies and social, physical, economic and environmental levers for change across increasingly obesogenic environments [3–6].

The transportation sector is increasingly being recognised for its potential contribution to improving population levels of incidental PA. Active transport (AT, defined as walking, cycling and use of public transport) reduces the risk of all-cause mortality [7] and cardiovascular disease [8] and may deliver other significant health and environmental co-benefits [9]. Rates of car ownership have dramatically increased worldwide in recent decades. Many countries, such as Australia, are highly car dependent with low prevalence of AT (for example, in Australia only 2% of the employed population in 2012 cycled to work, and 4% walked) [10]. Recent studies have suggested associations between transport mode and obesity, with more active forms of transport being negatively associated with measures of adiposity [11–14]. Whilst evidence on the obesity effect of modal choice is currently relatively limited [15, 16], interventions that encourage more active forms of transport may offer potential as population health strategies for obesity prevention.

The complexities of changing transport behaviours and re-engineering car-centric environments are however large. Transport behaviours are influenced by a wide range of factors, including the characteristics of travel modes (for example cost, availability, ease, comfort), individual influences (for example age, gender, income, physical ability) and contextual factors (for example culture, the built environment, climate, topography) [17]. Whilst it is recognised that a combination of legal, economic, communication and physical approaches to intervention will most likely be required to encourage modal shift [18], the reality is that very little is currently known about the effectiveness and cost-effectiveness of specific transport interventions when incorporating health, environmental and other effects [19, 20].

This paper seeks to conduct a scoping review and cost-effectiveness modelling study of a specific transport intervention that may encourage modal shift to more active forms of transport - an increase in automotive fuel excise taxation. To date, limited studies have been conducted into the potential effect of changes in fuel taxation on health related behaviours. A review by Mozaffarian et al. [21] used a Delphi approach and found that the evidence of effect for increasing participation in AT by raising fuel prices was not well-established but that the intervention might be considered. The review by Martin et al. [22] suggested that financial incentives, including the negative financial incentive of increased fuel price, may play a role in increasing PA however more rigorous evidence is required to make better use of effectiveness evidence in resource allocation decision-making. In 2013, Dhondt et al. [23] undertook the only study quantifying the health impact of an increase in fuel price published to date, but included only mortality related health benefits of an increase in walking and cycling and not morbidity-related health impacts. Our study therefore contributes to this literature by synthesising the current body of evidence for an obesity-related health effect of fuel excise taxation and conducting a scenario analysis detailing potential health gains and cost-effectiveness of a change in policy.

## Methods

**Fig. 1:**
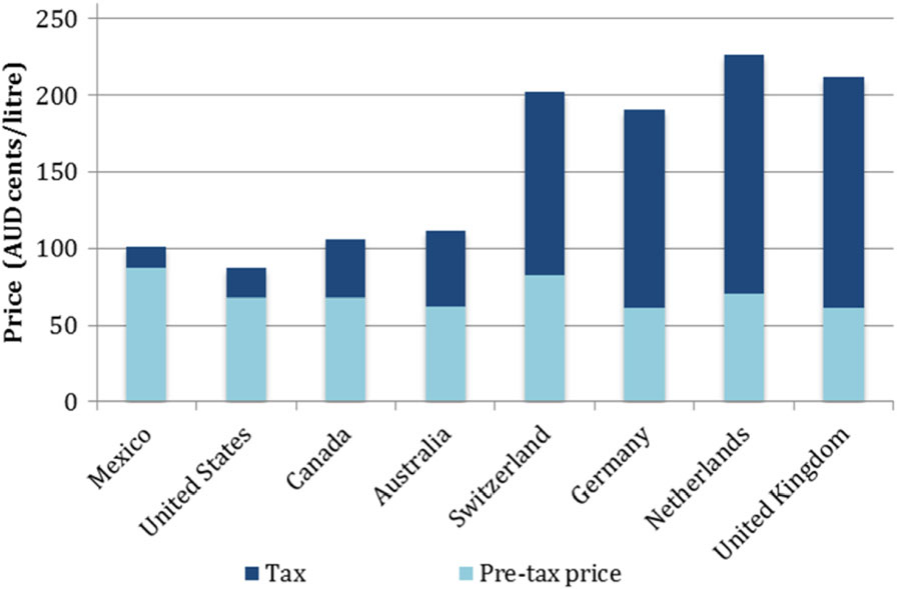
Fuel price for selected OECD countries, March quarter 2016. AUD=Australian dollars. 1 AUD equals approximately 0.74 US dollars or 0.59 British pounds as of November 2016. Source: Australian Government Office of the Chief Economist [35]

Taxation on fuel is common worldwide. Excise duty is a tax levied on alcohol, tobacco and fuel and petroleum products produced, stored or manufactured in Australia. The excise provides a general source of revenue to the Australian government [24]. Australia currently has the fourth lowest automotive fuel price of Organisation for Economic Co-operation and Development (OECD) countries [25]. The proportional amount of tax levied on Australian fuel is also low in comparison to almost all other OECD countries (Fig. 1), and has been decreasing in recent years [26].

**Fig. 2:**
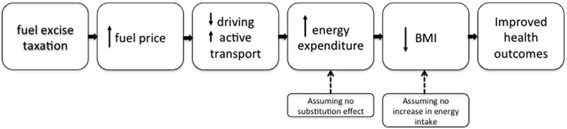
Logic pathway between increase in fuel excise taxation and improved obesity and PA-related health outcomes

By increasing the relative cost of driving to the motorist, government intervention to increase fuel excise taxation may present a feasible logic pathway to encouraging more AT across populations (Fig. 2). The price signal may lead to increased walking, cycling and use of public transport (PT). This in turn may lead to an increase in energy expenditure (assuming that PA-related behavioural substitution does not occur), a change in body mass index (BMI) (assuming there is no corresponding change in energy intake) and improved obesity and PA-related health outcomes.

A scoping review was therefore undertaken to explore the evidence and to inform parameters for cost-effectiveness modeling of an intervention to increase fuel excise taxation. The premise for the increase in tax in this paper is based on its potential to improve rates of AT. The scoping review consisted of two parts, described below.

### Scoping review of published associations between obesity, PA, walking or cycling and fuel price or taxation

A scoping review of the evidence for PA or obesity effect of motor vehicle fuel price or taxation was undertaken in May 2016. Given the scoping nature of the search, one reviewer (VB) designed and undertook the search strategy, whilst the second reviewer (RC) verified the strategy and resultant inclusions. The EBSCOHost and Web of Science databases were searched for papers reporting associations between fuel price or taxation and obesity, PA, walking or cycling effects. The full search strategy is given in Appendix 1. Study aims, methods and results were extracted and study quality was assessed by one reviewer (VB), using criteria based on the Strengthening of Reporting of Observational Studies in Epidemiology (STROBE) guidelines [27] and criteria adapted from previous studies [14, 28] (Appendix 2).

### Scoping review of published cross price elasticities of public transport demand for the Australian context

Given that PT accessibility predominantly relies on walking trips [29, 30], studies reporting on the cross price elasticity of PT may also be relevant when examining obesity-related effects of transport policy. Cross price elasticity is defined as the responsiveness of the quantity demanded of one good to a change in the price of another good [31]. Cross price elasticities of demand for PT with respect to fuel price may be context dependent [32]. Therefore a scoping review of published estimates of cross price elasticity of demand for PT with respect to fuel price suitable for the Australian context was conducted.

The Australian Government Bureau of Infrastructure, Transport and Regional Economics (BITRE) maintains a transport elasticities database that is freely and publicly available [33]. A search was conducted of all tables listed in the database reporting values of cross price elasticity for PT with respect to fuel price. A non-systematic search was then conducted for reviews or meta-analyses reporting estimates. The EBSCOHost and Web of Science databases were searched using key terms, including “public transport*”, “transit”, “meta-analysis”, “review”, “systematic review” and “elasticit*”. A search of the grey literature was also undertaken to capture any potential inclusions from other government or non-peer reviewed sources. The full search strategy is given in Appendix 1.

### Data selection and cost-effectiveness modelling

Evidence from the scoping review was used to conduct scenario analyses of the cost-effectiveness of a fuel excise taxation increase for the Australian population, incorporating both mortality and morbidity effects. The intervention was defined as an AUD0.10 per litre increase to the national fuel excise tax [34], which as of June 2010 was AUD0.38143 cents per litre [34]. An increase in excise of AUD0.10 would mean that the proportional amount of tax levied as a percentage of total fuel price would be higher, but still less than in countries such as Switzerland, the Netherlands and the United Kingdom [35]. The increase in excise was assumed to apply prior to the addition of the 10% Goods and Services Tax (GST), and it was assumed that the economic incidence of the increased tax was borne by the consumer (a realistic assumption given the relatively price inelastic nature of automotive fuel in Australia [36, 37]).

A proportional multi-state, multiple cohort life table model estimated obesity and PA-related health outcomes for the 2010 Australian population. Key model variables are listed in Table 1. Health outcomes were modelled as the difference between: (i) the 2010 reference year Australian population BMI and PA distributions; and (ii) the intervention population, which was identical to the reference population but incorporated changes to BMI and PA attributable to the intervention. Data on transport behaviours are not comprehensively collected in Australia at the national level, however the five-yearly Australian Bureau of Statistics Census of Population and Housing collects reliable data on the method of transport to work [38]. Therefore the intervention population was defined as the working age population (aged 18 to 64 years) and the impact of commuting modal switch as a hypothetical result of the intervention was estimated.

**Table 1:** Key model variables, mean value and 95% uncertainty intervals

Variables					Data source
Total population estimates (population numbers, mortality rates, BMI distribution)					ABS Census 2011 [38]
Disease epidemiology, relative risks, disability weights, total years of life lived with disability					GBD 2010 [43]
Disease healthcare costs					AIHW 2004 [45]
Transport mortality data					Australian Road Deaths Database [98]
Transport morbidity data					Henley et al. 2012 [99, 100]
Variables	Mean values and 95% UI^a^ (where applicable)				Data source and assumptions
Prevalence of using public transport for commuting purposes	*Males*		*Females*		ABS Census 2011 [38]
	18y	4.5%	18y	6.9%	
	19y	5.8%	19y	8%	
	20-24y	8.5%	20-24y	11.1%	
	25-29y	11.7%	25-29y	13.1%	
	30-34y	11.1%	30-34y	9.9%	
	35-39y	9.1%	35-39y	6.8%	
	40-44y	7.4%	40-44y	5.9%	
	45-49y	6.3%	45-49y	5.7%	
	50-54y	5.8%	50-54y	5.3%	
	55-59y	4.9%	55-59y	4.5%	
	60-64y	3.3%	60-64y	2.9%	
Cost of legislation (including RIS process)	AUD1,090,792 (95% UI AUD939,805–1,249,710)				Sampled from a gamma distribution, taken from estimates from Lal et al. [49].
ABS average weekly earnings	AUD1,241 (95% UI AUD1,126–1361)				Sampled from a gamma distribution (mean 1530.20, s.e. 44.8) Professional, Scientific and Technical Services full time adult average half-hour time cost and 14% labour oncosts, from Government sources [48, 50, 51].
Number of businesses affected	185,959 (95% UI 172,747–199,317)				Sampled from a pert distribution (most likely = 186,097) quoted from Government source, +/−10% [48].
Total intervention cost	AUD4,381,691 (95% UI AUD3882,683–4,903,984)				

*Table notes:* ^a^95% uncertainty interval (UI) based on 2000 simulations. *ABS* Australian Bureau of Statistics, *AIHW* Australian Institute of Health and Welfare, *AUD* Australian dollars, *GBD* Global Burden of Disease, *RIS* Regulatory Impact Statement, *s.e* standard error

The multi-state life table method incorporated disease-specific lifetables to estimate mortality and morbidity for nine obesity-related diseases and five overlapped PA-related diseases (ischaemic heart disease, hypertensive heart disease, ischemic stroke, diabetes, colorectal cancer, kidney cancer, breast cancer, endometrial cancer and osteoarthritis). Modal shift to more active forms of transport may also change the risk of injury from transport accidents. The ‘risk injury matrix approach’, as proposed by Bhalla et al. [39] and used in several health impact assessments [40–42], was adapted to estimate the change in absolute numbers of mode-specific fatalities and serious injuries as a result of the intervention. Estimates were then incorporated into the lifetable modelling, and compared with baseline mode-specific road traffic accident deaths and years lived with disability (YLD) from the Global Burden of Disease (GBD) study 2010 [43]. PA effect from the intervention was modelled using effect estimates from the scoping review and relevant input parameters. To ensure conservative results, any uptake in PA as a result of the intervention was assumed to have occurred in those already moderately or highly physically active in their daily lives. PA effect was modelled to BMI effect using the energy balance equation by Hall et al. [44] (Appendix 3). In the absence of evidence on the long-term effects of fuel price increases on public transport use, we assumed that the behavior change would be sustained (i.e. that those who switched to public transport continued to use public transport over the long-term).

Data on healthcare costs were obtained from the Australian Institute of Health and Welfare (AIHW) for 2001 due to data availability [45], and inflated to 2010 prices using the Health Price Index [46]. Intervention costs were regarded as minimal given that: (i) fuel excise taxation, with bi-annual indexation, already occurs within Australia; and (ii) the excise is levied at the point of production or import and there are relatively few producers/importers of transport fuels in Australia. It is therefore expected that the administrative and compliance burden of the tax would be relatively low [47]. Intervention costs were estimated using information from an Australian Government Regulatory Impact Statement [48] and publicly available data on wage costs and use of parliamentarians time [48–51] Costs and cost savings were discounted at 3% and all values are reported in AUD2010 dollars (Table 1).

Economic evaluations of transport interventions differ in their inclusions of other potential cost or cost saving categories [20]. Travel time savings and car parking cost savings are difficult to generalise given the large scope for variation in costs when modelling nationally, and therefore have not been included in our analysis. Decongestion and environmental benefits are also difficult to generalise on a national basis and therefore have also been omitted. Vehicle operating cost (VOC) savings related to fuel and repairs and maintenance were estimated using conservative parameter values from Australian guidelines [52] and reported separately as potential replacement expenditure per new active traveller (i.e. as resource corrections between automobile and PT usage costs). We assume that those new to AT will continue to own and use private motor vehicles for other purposes. For consumers changing their travel behaviours in response to financial incentives, the ‘rule of half’ was applied to VOC savings as per national guidelines [52]. The ‘rule of half’ is based on the economic theory that when consumers change their travel in response to a financial incentive, the net consumer surplus is equivalent to half of their price change [53].

Health adjusted life years (HALYs) gained were estimated by comparing the intervention to the ‘do-nothing’ comparator. A limited societal perspective was adopted, with the time horizon for estimating cost offsets and HALY benefits being rest-of-life or 100 years. Incremental cost-effectiveness ratios (ICERs) were calculated by dividing the difference in the net cost of the intervention by the difference in the net health benefit. ICER results are presented on a cost-effectiveness plane, where interventions that are both cost saving and of benefit to health are considered ‘dominant’. Interventions falling in the other quadrants of the plane will be determined as cost-effective using the AUD50,000 per HALY threshold as per Australian benchmarks [54].

All modeling was undertaken using Excel 2010, with uncertainty analysis around the relative risk of incident disease and key input parameters estimated by Monte Carlo simulation using the Excel add-in Ersatz (version 1.34) [55]. For input parameters with considerable uncertainty, we have adopted a conservative approach to estimation of potential cost-effectiveness. One-way sensitivity analyses were then undertaken to test the validity of assumptions and robustness of results [56]. We also present “plausible case” scenario results, using higher but still credible values as an indication of the potential range for cost-effectiveness. Parameters for sensitivity analyses are given in Appendix 4.

Consideration of the broader impacts of an intervention should also be considered alongside any cost-effectiveness analysis, in order to take into account factors that are important to decision-makers but difficult to quantify within the analysis [3, 56]. Cost-effectiveness results are therefore discussed alongside a “second stage filter analysis”, which analyses potential equity, feasibility, acceptability and sustainability effects of the intervention.

## Results

### Results from the scoping review of published associations between obesity, PA, walking or cycling and fuel price or taxation

**Fig. 3:**
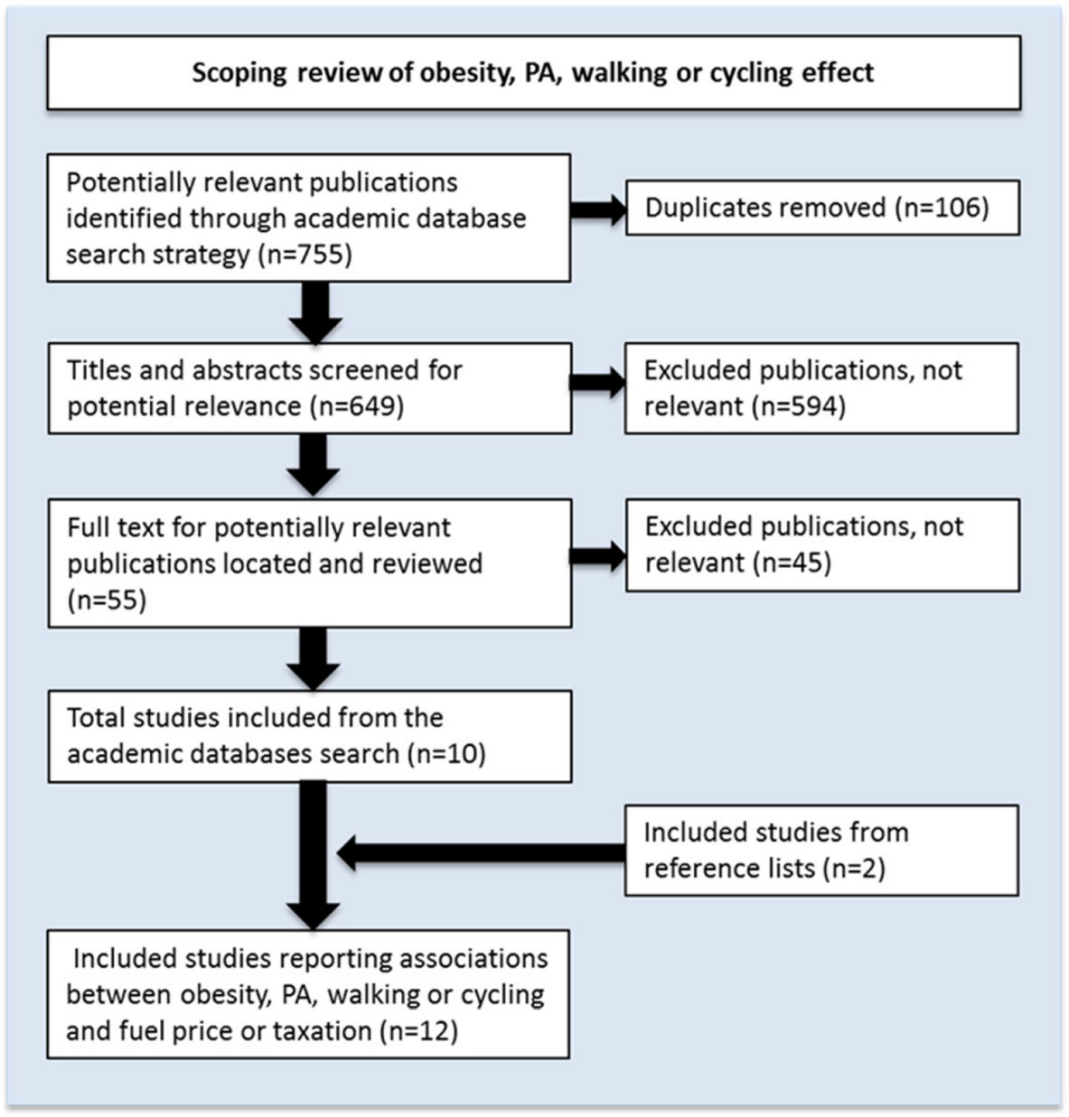
PRISMA flowchart of included studies

A total of 12 studies were included in our evidence review of obesity, PA, walking or cycling associations with fuel taxation or price (Fig. 3). Limited evidence currently exists in the peer-reviewed literature on the effect of fuel price or taxation on obesity specifically, with only three primary studies found [57–59] (Table 2). All three studies were cross-sectional study designs, and only the study by Courtemanche [57] examined individual level effects.

**Table 2:** Studies reporting associations between fuel taxation or price and obesity, PA, walking or cycling

Study	Location/Population	Study aim	Method	Variable of interest (Outcome)	Relevant findings	QA
*Obesity*						
Courtemanche 2011 [57]	USA Adults (*n* = 1,807,266)	To estimate the effect of fuel price on weight and obesity, by looking at its effect on PA, frequency of eating at restaurants and food choices at home.	Cross-sectional	Fuel price (BMI (S))	USD2004 $1 increase in fuel price reduces BMI by approx. 0.35 units (s.e. 0.050, *p* < 0.01). A permanent USD1.00 increase in fuel prices would, after 7 years, reduce U.S. overweight prevalence by approx. 7% and obesity by approx. 10%.	8
Rabin et al. 2007 [58]	24 European countries	To describe obesity patterns and examine macro-environmental factors associated with obesity prevalence.	Ecological, cross-sectional	Fuel price (Prevalence of obesity using BMI (S))	The price of fuel was associated with obesity prevalence for females (b = −0.096, *p*-value 0.041) and overall (b = −0.095, *p*-value 0.0542), but not for men.	5
Sun et al. 2015 [59]	47 low-middle income countries	To identify CVD risk factors in low-middle income countries.	Ecological, cross-sectional	Fuel price (Prevalence of obesity using BMI (S))	The price of fuel was not statistically significantly associated with obesity in either men or women.	5
*Physical activity*						
Hou et al. 2011 [60]	Birmingham, Chicago, Minneapolis and Oakland, USA. Young adults 18–30 years at baseline (*n* = 5115)	To investigate longitudinal associations between fuel price and physical activity.	Longitudinal cohort	Fuel price (Leisure PA - energy units (S))	A hypothetical USD0.25 increase in fuel price significantly associated with increase in energy expenditure (9.9 energy units (EU), 95% UI: 0.8–19.1, *p*-value 0.03). Equivalent to an increase in walking per week of 17 min. After controlling for all covariates, an USD0.25 increase in fuel price was associated with 1.3 EU increase in walking score (*p* = 0.2), equivalent to an additional 3 min of walking per week. Results suggest relatively weak association between fuel price and walking. No significant association for cycling.	7
Sen 2012 [61]	American adults 15 years plus (*n* = 81,957)	Uses data from the time of fuel price rises due to Hurricane Katrina to explore effect on PA.	Cross-sectional	Fuel price (PA, defined five ways: (1) walking, running, bicycling or rollerblading as part of LTPA, (2) walking or cycling to work or errands, (3) playing with kids, (4) housework of MET > = 3, (5) total time spent on all PA MET > =3. (S))	Higher fuel prices show some association with increases in LTPA (sig. at *p* < 0.05). Walking and bicycling to work or errands statistically weak and sensitive to model specification. Only one approach resulted in *p* < 0.05 with OLS estimate 0.74. Changes in participation and time spent in walking or cycling are not large. No association was found between higher fuel prices and PT use, although may be due to lack of accessibility to PT.	8
Sen et al. 2014 [62]	American high school students grades 9–12 (*n* = 58,749)	To examine the relationship between fuel price and driving behaviours in teens.	Cross-sectional	Fuel price (moderate PA, defined as: (1) whether participates in PA “that did not lead to sweating or breathing hard”, and (2) whether participates more than five times per week or not (S)).	Higher fuel prices positively associated with higher levels of moderate PA. Higher fuel prices associated with moderate PA at least 1 day of the week for females (ME = 3.25%, t-stat = |2.90|), males (ME = 2.32%, t-stat = |2.36|), other races (ME = 3.01%, t-test = |2.16|), and teens ages 16 years and younger (ME = 3.98%, t-stat =14.70|). Higher fuel prices were associated with frequent moderate PA for females (ME = 1.92%, t-stat = |2.19|), males (ME = 3.63%, t-stat = |4.16|), non-Hispanic whites (ME = 3.88%, t- stat = 12.511), other races (ME = 3.85%, t-stat = |2.27|), and teens ages 16 years and younger (ME = 3.54%, t-stat = |4.54|).	7
*Cycling*						
Buehler & Pucher 2012 [63]	USA, population of 90 cities	To examine the association between levels of cycling and cycle infrastructure.	Cross-sectional	Fuel price (share of workers commuting by cycling (S))	State fuel prices had a significant positive correlation with cycling levels (correlation coefficient 0.5, sig. at 95%), consistent with the theory that higher fuel prices may lead to more cycling to work.	5
Dill & Carr 2003 [68]	USA, population of 35 large cities	To explore associations between cycling infrastructure and cycling.	Cross-sectional	Fuel price (share of workers commuting by bicycle (S))	Although results on fuel price were not explicitly reported, authors state that fuel price was not statistically significant.	4
Pucher & Buehler 2006 [67]	USA/Canada Population of 18 cities	To explore higher cycling rates in Canada than US.	Cross-sectional	Fuel price (share of workers commuting by cycling (S))	Higher fuel prices are associated with higher rates of cycling to work (coefficient 3.040 (s.e. 1.159, significant at 95% level, adjusted R^2^ 0.596).	6
Rashad 2009 [64]	USA, metropolitan area residents (BRFS *n* = 146,730 NPTS *n* = 73,903)	To determine the relationship between cycling and fuel price.	Cross-sectional	Fuel price (cycling, defined as (1) cycled for pleasure in past month or (2) cycled in a trip yesterday (S))	Increasing fuel price by $1 increased the probability of cycling by between 1.6% (t-stat 3.30, *p* < 0.01) and 4.7% (t-stat 2.17, *p* < 0.05) for men. Results for women ranged between 1% (t-stat 5.11, *p* < 0.01) and 3.5% (t-stat 3.05, *p* < 0.01).	7
Smith & Kauermann 2011 [65]	Residents of Melbourne, Australia	To examine the determinants of cycling, including the cross-price elasticity of cycling.	Cross-sectional	Fuel price (cycling volumes (O))	Substitution into cycling as a mode of transport observed in response to increase in fuel prices, particularly during peak commuting periods and by commuters originating in wealthy and inner city neighbourhoods. Cross-price elasticities vary depending on loop data and method used and time of day, from approximately 0.18 to 0.48 during peak commuting periods, significant at either 5% or after Bonferroni adjustment.	7
*Walking*						
Ryley 2008 [66]	West Edinburgh, adults living in West Edinburgh who drive (*n* = 627)	To estimate propensity for motorists to walk for short trips, based on changes to fuel price, journey time, parking costs.	Cross-sectional, discrete choice modelling, using stated preference data from survey	Fuel price (propensity to walk (SP))	Fuel coefficient − 0.4159, significant at 5% (t-value −6.3). Fuel price had lower relative influence than parking costs, or journey time.	4

*Table notes*: *BMI* body mass index, *DALYs* disability adjusted life years, *EC* elemental carbon, *EU* energy units, *LTPA* leisure time physical activity, *ME* marginal effect, shows the percentage point change in the probability of the outcome being 1, *MET* metabolic equivalent task, *(O)* objectively measured, *OLS* ordinary least squares, *PA* physical activity, *PT* public transport, *QA* quality assessment, *(S)* self-report, *s.e.* standard error, *(SP)* stated preference, *UI* uncertainty interval, *USD* United States dollars, *VKT* vehicle kilometres travelled, *VMT* vehicle miles travelled

Nine studies specifically explored the association between fuel taxation or price and PA, walking or cycling [60–68](Table 2). The majority of these studies (89%) were cross-sectional [61–68]; with only one longitudinal study investigating the relationship between fuel price and leisure PA [60]. Over half of these studies (55%) reported specifically on associations between fuel taxation or price and cycling [63–65, 67, 68], perhaps reflective of the increasing evidence of the positive health benefits of cycling for utilitarian purposes [69].

The mean quality assessment score of all included studies was relatively low (6 out of a possible score of 10, range 4 to 8) (Appendix 5), and is partly attributable to the use of cross-sectional study designs and self-reported outcomes. The challenges of collecting rigorous evidence of effect for environmental or policy-type interventions are well-recognised, with calls for better use of ‘natural experimental’ designs and a more pragmatic approach to the traditional hierarchies of evidence for interventions not amenable to evaluation through controlled circumstances [70, 71].

### Results from the scoping review of published cross price elasticities of PT demand

Estimates of cross price elasticity of demand for PT with respect to fuel price vary due to geographical location, time and modal share, but also due to model specification, statistical methods and quality of data used [72, 73]. Our scoping review found eight relevant studies reporting on cross price elasticity of PT demand with respect to fuel price. Two estimates of cross price elasticity were found from the search of the BITRE database [33], and six estimates were found from our non-systematic search [73–78] (Table 3).

**Table 3:** Estimates of cross price elasticity of demand for PT with respect to fuel price, focusing on Australian values

Source	Type of study	Estimate
BITRE database [33], Table 1D03	Cited from study by Goodwin [101]	0.34
BITRE database [33], Table 2D18	Cited from studies by Cervero 1990 and Wang & Skinner 1984 (*further details not given*)	0.08 to 0.80
Currie & Phung 2006 [74]	Review within primary study	0.07 to 0.8
Currie & Phung 2008 [75]	Review within primary study	LR: 0.07 to 0.30
Holmgren 2007 [78]	Review Meta-analysis	0.38 (s.e 0.31) SR: 0.82 (95% UI 0.56 to 1.08) LR: 1.15 (95% UI 0.65 to 1.65)
Kennedy & Wallis 2007 [76]	Review	0 to 0.20
Litman 2016 [73]	Non-systematic review	SR: 0.05 to 0.15 LR: 0.2 to 0.4
Luk & Hepburn 1993 [77]	Review	SR: 0.07

*Table notes: BITRE* Bureau of Infrastructure, Transport and Regional Economics, *LR* long-run, *s.e* standard error, *SR* short-run, *UI* uncertainty interval

Overall, limited consensus exists on values for either short-run or long-run cross price elasticity of demand for PT with respect to fuel price. The study by Currie & Phung [74] cited cross price elasticities within a probable range of 0.07 to 0.80, although authors noted the wide range and potential for variability from use of these estimates. In a subsequent paper, the authors estimated variability between different Australian cities at different times of day and between different PT modes, finding that variations in service levels, infrastructure and peak vs. non-peak travel may explain some differences [75].

The review by Kennedy & Wallis [76] found that cross price elasticities for rail services may be higher than those for other PT modes, citing a range of 0.48 to 0.80 for rail and recommending lower cross price elasticities for general PT services from around zero to 0.20. The review by Luk & Hepburn [77] recommended a short-run cross price elasticity of 0.07. The updated review by Litman [73] recommended cross price elasticity values of between 0.05 and 0.15 in the short-run and between 0.2 and 0.4 in the long-run, incorporating Australian studies into the analysis of this probable range.

Holmgren [78] conducted a meta-analysis of bus demand elasticities and estimated the expected values for cross price elasticity of demand with respect to petrol price for Australia as 0.82 in the short-run (95% UI 0.56–1.08) and 1.15 in the long-run (95% UI 0.65–1.65). The author suggested that estimates were much higher for Australia than Europe (short-run cross price elasticity in Europe 0.4 (95% UI 0.16–0.64), long-run cross price elasticity in Europe 0.73 (95% UI 0.38–1.08)). Estimates from the meta-analysis are also obviously much higher than other studies reported here (Table 3). The mean cross price elasticity of demand for PT with respect to fuel price across all 17 included studies by Holmgren 2007 [78] was 0.38 (s.e 0.31), although no detail was given on the search method or inclusion criteria for studies and references of the included studies were not cited.

### Results from cost-effectiveness modelling

Due to the relatively limited evidence of obesity, PA, walking or cycling effect (Table 2), cost-effectiveness scenarios of an increase in fuel taxation were modelled using conservative estimates of cross price elasticity of demand for public transit with respect to fuel price and key input parameters. To avoid over-estimating potential health benefits, we selected the conservative cross price elasticity value of 0.07, with potential health benefits resulting from the increase in walking to access PT as the basis for our hypothetical main scenario. Key input parameters for estimation of intervention effect are given in Table 4.

**Table 4:** Input parameters for estimation of intervention effect, mean value and 95% uncertainty intervals

Parameter	Mean values and 95% UI^a^ (where applicable)	Sources and assumptions
Cross price elasticity for PT with respect to fuel price	0.07	Derived increase in the prevalence of PT commuting of 0.61% [38]. Modelled to PA/BMI effect (Appendix 3). Assumed all new PT users were previous car drivers, a reasonable assumption given the high prevalence of driving to work in Australia [38].
Average annual retail fuel price (national, metropolitan) (cents per litre)	125.39 cents (95% UI 124.95–125.86)	Sampled from a gamma distribution, from national metropolitan fuel price [102].
Marginal MET^b^ value for walking to access PT	2.5 (95% UI 0.7–6.4)	MET value for walking to access PT 3.5 from Ainsworth et al. 2011 [81], adjusted for inactivity. Sampled using a lognormal distribution (stdev 1.6 from Gotschi et al. 2015 [103]).
Average distance a person will walk to access PT (metres)	400	Based on ‘rule of thumb’ planning guideline for distance walked to bus/tram access points [104, 105].
Comfortable gait speed (cm/s)	*Males* 18-29y = 139.2 (95% UI 110.5–172) 30-39y = 145.7 (95% UI 128.4–164.2) 40-49y = 145.6 (95% UI 115.6–180.4) 50-59y = 139.5 (95% UI 100.5–192.2) 60-64y = 136.3 (95% UI 100.9–179.7) *Females* 18-29y = 140.3 (95% UI 109.3–177.4) 30-39y = 140.8 (95% UI 117.5–166.9) 40-49y = 139.2 (95% UI 111.5–172.1) 50-59y = 139.5 (95% UI 112.2–170.8) 60-64y = 129.6 (95% UI 90.8–172.7)	Sampled from a lognormal distribution, taken from estimates from Bohannon 1997 [106]. Using average distances and gait speeds this results in an average increase in walking to access PT of 18.9 min per day in men and 19.2 min per day in women. This falls within the range summarised by Rissel et al. [30] of 8 to 33 min PA associated with PT use.
Number of weeks of intervention effect (averaged over year)	49 (95% UI 46–52)	Sampled from a uniform distribution based on estimate of number of working weeks per year for full-time workers.

*Table notes:* ^a^95% uncertainty interval (UI) based on 2000 simulations. ^b^ = Metabolic equivalent task (MET) value defined as the ratio of activity specific metabolic rate to standard resting metabolic rate of 1.0 [81]. *ABS* Australian Bureau of Statistics, *AUD* Australian dollars, *cm/s* centimetres per second, *PA* physical activity, *PT* public transport, *RIS* regulatory impact statement, *SA* sensitivity analysis, *VISTA* Victorian Integrated Survey of Travel and Activity, *Y* years of age

**Fig. 4:**
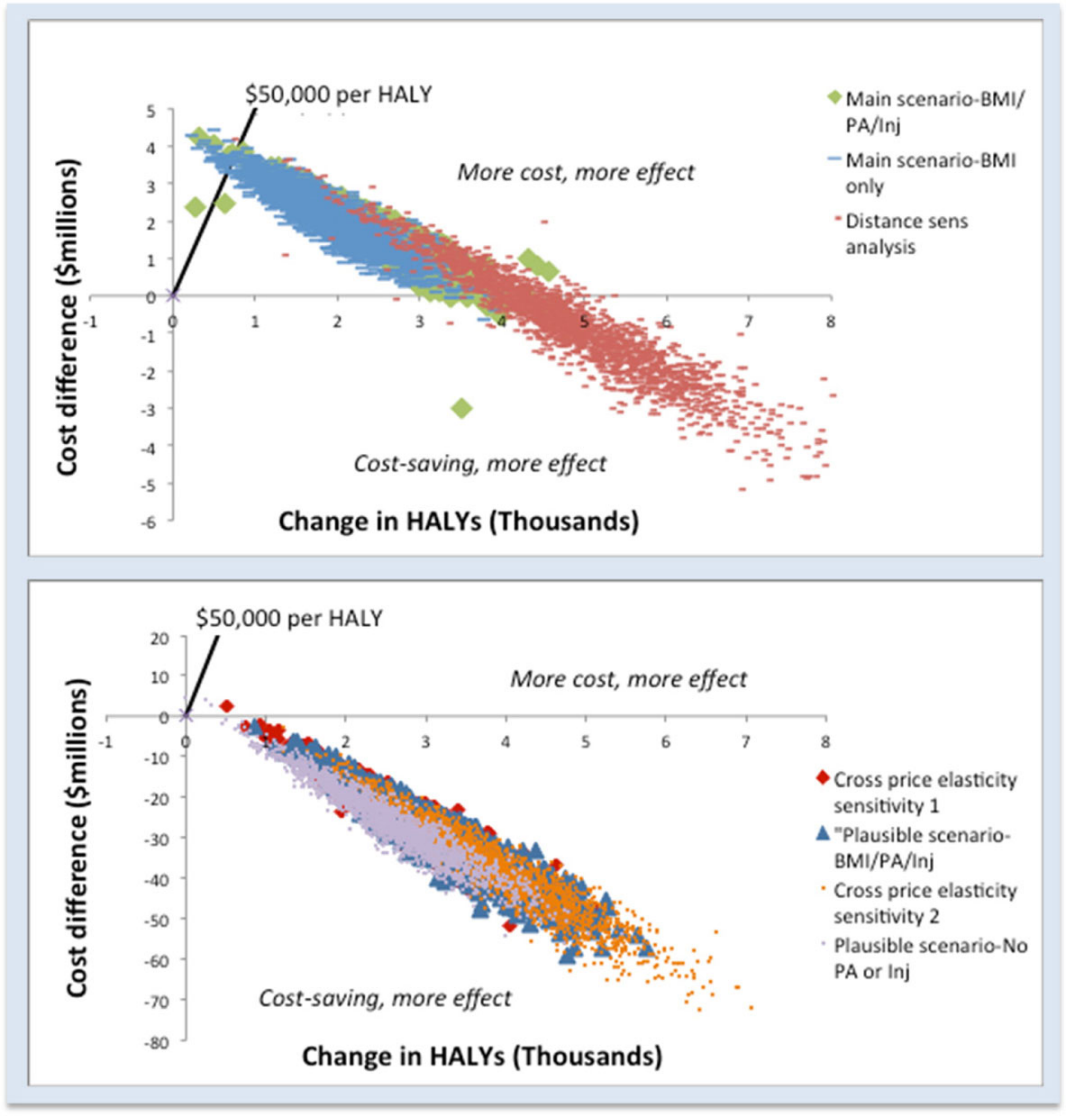
Cost-effectiveness planes, net cost per HALY saved

Based on our conservative modelling inputs and assuming effect stability over the lifetime, 237 HALYs would be gained as a result of the intervention (95% UI 138–351). A total of AUD2.6 M in healthcare costs would be averted (95% UI AUD1.3 M–3.9 M)(Table 5). The intervention would result in an overall decrease in mortality and morbidity from traffic accidents (3 deaths averted and 79 years lived with disability (YLDs) averted). The ICER suggests that the intervention would be cost-effective over the lifetime, with a median ICER of 7702 (95% UI 1366–22,125). The probability of the intervention being dominant (cost saving) however is only 0.8% (Table 5, Fig. 4).

**Table 5:** Results, main scenario and sensitivity analyses

*Results per scenario*		Total HALYs saved	Total healthcare cost savings (AUD 2010)	Net cost per HALY saved (with cost offsets) (ICER, AUD 2010)
Main scenario	Main scenario BMI/PA/injury effect	237 (95% UI 138–351)	$2,552,925 (95% UI $1,304,017–$3,905,568)	$7702 saved per HALY (95% UI $1366–$22,125) (Probability of cost-effectiveness 99.7%) (Probability of cost-saving 0.8%)
	Main scenario BMI effect only	195 (95% UI 85–314)	$2,310,366 (95% UI $962,352–$3,762,993)	$10,514 saved per HALY (95% UI $1843–$39,990) (Probability of cost-effectiveness 98.4%) (Probability of cost-saving 0.3%)
One-way sensitivity analyses	Cross price elasticity 1 (0.82 from Holmgren [78])	2769 (95% UI 1614–4056)	$29,928,506 (95% UI $15,124,893–$45,413,548)	Dominant (Probability of cost-effectiveness 100%) (Probability of cost-saving 99.95%)
	Cross price elasticity 2 (1.15 from Holmgren [78])	3882 (95% UI 2233–5714)	$42,000,179 (95% UI $20,713,001–$63,854,358)	Dominant (Probability of cost-effectiveness 100%) (Probability of cost-saving 100%)
	Distance walked 800 m	472 (95% UI 258–705)	$5,098,746 (95% UI $2,422,181–$7,810,093)	Dominant (Probability of cost-effectiveness 99.95%) (Probability of cost-saving 71%)
“Plausible case”	“Plausible case” scenario – BMI/PA/injury effect	3181 (95% UI 1797–4633)	$34,239,586 (95% UI $17,433,480–$51,336,591)	Dominant (Probability of cost-effectiveness 100%) (Probability of cost-saving 100%)
	“Plausible case” scenario – BMI only	2532 (95% UI 1084–4098)	$30,222,697 (95% UI $12,875,579–$47,444,286)	Dominant (Probability of cost-effectiveness 100%) (Probability of cost-saving 99.9%)

*Table notes:* Reported values are medians. *AUD* Australian dollars, *HALY* health adjusted life year, *95% UI* 95% uncertainty interval, *BMI* body mass index, *PA* physical activity, *ICER* incremental cost-effectiveness ratio, *MET* metabolic equivalent task, *m* metres

If we model only health-related costs and benefits of diseases related to obesity (and omit the independent effects of diseases related to PA and mortality and morbidity from the change in risk of injury) 195 HALYs would be gained over the lifetime (95% UI 85–314), with AUD2.3 M in obesity-related healthcare cost savings (95% UI AUD0.96 M–3.8 M). If only considering the obesity-related effects the intervention is still considered cost effective with a median ICER of 10,514 (95% UI 1843–39,990), however the probability of the intervention being dominant is only 0.3% (Table 5, Fig. 4).

One-way sensitivity analyses were undertaken to test the robustness of results to variations in some of the key input parameters, especially those where uncertainty analysis was not possible due to data constraints (Table 5) (Appendix 4). Our cost-effectiveness results are very sensitive to the choice of cross price elasticity used (Table 5). Both sensitivity analyses varying cross-price elasticity result in far higher HALY and healthcare cost saving estimates and the intervention is dominant. The burden of morbidity and mortality from traffic accidents is also reduced in both scenarios (14 deaths and 356 YLDs averted (*cross price elasticity 1*); 20 deaths and 501 YLDs averted (*cross price elasticity 2*)).

Evidence also suggests that people will walk more than 400 m to access trains in particular, with 800 m regarded as the planning “rule-of-thumb” and some research demonstrating that 800 m may still be a conservative estimate for train accessibility [79, 80]. If we vary the distance walked to access PT to 800 m in our main analysis the intervention is also dominant (Table 5). Increased distance to access PT would however result in an increase in mortality from traffic accidents (5 fatalities gained) but a decrease in morbidity (33 YLDs averted) due to the change in traffic exposure. The use of 3.5 METs for walking is also relatively conservative when estimating walking for commuting purposes [81]. Obviously use of a higher MET value for walking to access PT would also result in greater health benefits and cost-effectiveness.

Due to the fact that many of the input parameters for our main analysis are relatively uncertain but based on conservative estimates, we modelled a “plausible case” cost-effectiveness estimate using the input parameters given in Appendix 4. If we assume higher ‘plausible case’ values for cross price elasticity, MET value for walking and distance walked 3181 HALYs would be gained over the lifetime (95% UI 1797–4633), with AUD34.2 M in healthcare cost savings from obesity and PA related diseases (95% UI AUD17.4 M–51.3 M). The intervention would be dominant (Fig. 4). If we model only health-related costs and benefits of diseases related to obesity for the “plausible case” scenario (and omit the independent PA and injury-related effects) 2532 HALYs would be gained over the lifetime of the cohort (95% UI 1084–4098), with AUD30.3 M in obesity-related healthcare cost savings (95% UI AUD12.9 M–47.4 M). If only considering these obesity-related benefits, the “plausible case” scenario is still dominant (Fig. 4).

Using Australian recommended values for vehicle operating cost (VOC) savings, we estimate that at least AUD689 could be spent per year on replacement trip costs per new PT user as resource correction costs (Table 6). As an indication of likely replacement trip costs, a metropolitan yearly train ticket in the state of Victoria cost the equivalent of AUD1,342 in the 2010 reference year for our analysis. It should be noted however that our conservative approach to estimating VOC savings likely results in underestimation (for instance, we have not included savings related to parking costs, oil or tyre replacement costs). If we assume even a AUD5.00 parking cost per day for full-time motor vehicle commuters for 46 weeks of the year the annual breakeven for replacement PT trip costs would be AUD1839 per new PT user (i.e. potentially an overall cost saving). Evidence suggests that central business district parking rates in Australia are in fact much higher [82].

**Table 6:** Cost savings per new person to PT as a result of the intervention

Cost or cost savings per new PT user	Values (AUD)	Source/Estimate
Vehicle operating cost (VOC) savings		
Annual petrol cost savings per new PT user (out-of-pocket cost savings for fuel saved)	$492.08	Annual distance (car driver km pp) saved, based on mean trip-stage distance (km) from home to workplace by car drivers from VISTA data [107] and verified by national data [108], full-time workers and national metropolitan fuel price [102]. ‘Rule of half’ applied.
Repairs and maintenance cost savings	$197.26	Annual distance (car driver km pp) saved, based on mean trip-stage distance (km) from home to workplace by car drivers from VISTA data [107] and verified by national data [108], full-time workers and National Guidelines [52]. ‘Rule of half’ applied.
VOC SAVINGS FOR THOSE NEW TO AT ^a^		$689
Including parking charges of $5 per business day^b^		$1839
Including parking charges of $10 per business day^b^		$2989
Including parking charges of $20 per business day^b^		$5289

*Table notes:* ^a^ only includes conservative parameters, therefore likely understimates potential cost savings. ^b^ based on full-time workers, for average 46 working weeks per year. *AUD* Australian dollars, *km* kilometres, *pp.* per person, *PT* public transport, *VISTA* Victorian Integrated Survey of Transport Activity

## Discussion

Despite the increasing awareness that AT may positively contribute to population levels of PA with resultant public health benefits, evidence on effective and cost-effective interventions to improve rates of AT is limited. Our hypothetical estimation of potential cost-effectiveness of a macro-level fuel excise taxation intervention incorporating both PA, injury and obesity-related health benefits therefore adds to the relatively limited evidence base on the potential for ‘upstream’ interventions for obesity prevention across populations [1]. To date, only one study has investigated the impact of a fuel price increase on health [23], finding that a 20% fuel price increase in Belgium resulted in relatively modest health benefits from reduced risk of mortality from physical inactivity, and reduced mortality and morbidity from injuries and emissions (1650 DALYs averted (95% UI 1010–2330)). Our results support these findings of a positive overall health impact of an increase in fuel price, and suggest that a fuel excise taxation intervention may be cost saving when including the PA, injury and obesity-related health benefits of a resultant increase in AT but that the magnitude of results is sensitive to relatively uncertain input parameters.

Active commuting could contribute substantially towards reaching the recommended Australian adult guideline levels for PA of 150 to 300 min of moderate intensity PA per week [83]. Our conservative estimates here suggest that walking to access PT for commuting purposes could increase walking for transport on average by 90 min per week (Appendix 3). This is within the range reported in the systematic review by Rissel et al. [30] of between 8 and 33 min of additional PA per day from walking to access PT, and further highlights the valuable contribution that incidental PA through AT could make to reducing population levels of diseases associated with physical inactivity. In order to produce conservative results we also assumed that the uptake in PA occurred in those already moderately or highly physically active. If the intervention encouraged those currently inactive to walk to access PT the potential health gains could be even greater but are likely to be less sustainable.

Modelling hypothetical PA effect to BMI effect using the validated approach by Hall et al. [44] suggests that small obesity-related health benefits are also achievable through AT policies and interventions. Our estimates (Appendix 3) fall within the range of plausible estimates from published studies. For example the longitudinal study by Martin et al. [12] estimating a BMI reduction from changing from commuting by private transport to AT of −0.32 kg/m^2^ (95% UI -0.60 kg/m^2^ to −0.05 kg/m^2^) or the longitudinal study by Flint et al. [84] estimating that middle age adults who commuted by AT had lower BMI than car-driving commuters (−1.0 kg/m^2^ in men (95% UI -1.13 kg/m^2^ to −0.84 kg/m^2^), −0.7 kg/m^2^ in women (95% UI -0.85 kg/m^2^ to −0.48 kg/m^2^)).

Our hypothetical main scenario results suggest modest health benefits using very conservative input parameters (237 HALYs, AUD2.6 M in healthcare cost savings over the lifetime). The range of results using less conservative but still credible inputs however suggests the potential for much larger population health gains (for instance, under our ‘plausible scenario’ 3181 HALYS gained and AUD34.2 M in healthcare cost offsets). Results demonstrate that a fuel excise taxation intervention could be cost effective from an obesity prevention perspective, with the median ICER from all of our analyses falling under the AUD50,000 cost-effectiveness threshold [54]. Results also highlight the importance of improving road safety for cyclists and pedestrians, with scenarios modelling increased distance to access PT resulting in higher mortality (although lower morbidity) and all other scenarios resulting in improved traffic related morbidity and mortality.

Findings from our scoping review and hypothetical modelling study demonstrate however the limitations and difficulties of collecting and modelling evidence on the health impacts of specific population level transport interventions. Until now health impact assessments and economic evaluations of active transport interventions have relied heavily on hypothetical scenario modelling and assumptions around effect to estimate costs and consequences [9, 20]. While the information that such studies provides is useful, now there is a need for actionable, implementable and effective ways to increase rates of AT across populations. Our scoping review however demonstrates just how difficult evaluating the public health credentials of non-health sector interventions is, given that relatively little empirical evidence currently exists on the impact of fuel taxation or price on AT behaviours. The limited scope of evidence currently available on obesity-related effect is somewhat expected, given that obesity is further along the causal pathway than PA effect when considering the impact of AT on health. However we also found relatively few studies examining associations between fuel price or taxation and walking and cycling specifically, coupled with the fact that limited consensus exists around cross price elasticities of demand for PT with respect to fuel price.

Our hypothetical modelling aimed to make the best possible use of the limited evidence base as it currently stands, however a lack of more rigorous evidence of effect is a limitation of our modelling. We have tried to circumvent this to the best of our ability by using conservative estimates of parameters and providing “plausible case” scenario analyses. It should be noted however that cross price elasticities may differ between different contexts or between different socio-economic groups (for instance urban residents who are better serviced by PT may be more cross price elastic than those who are not [85]); our modelling has not been able to incorporate any of this variation or complexity at this time. Use of a more direct and observable estimate of PA or obesity effect would be preferable for our economic modelling, and this is an area where significant scope for future research exists.

Anecdotally, some countries with high prevalence of AT also have high fuel prices (for instance, the Netherlands). Evidence suggests that transport policies that promote easy and relatively cheap access to a motor vehicle result in more kilometres of daily car travel [86, 87]. Logically, fuel price may be one of several contributing factors in deciding whether to engage in AT. Whilst many other contributing factors are also likely to exist, it is clear that a better understanding of the extent to which price levers such as fuel excise taxation might contribute to reducing obesogenic environments is warranted. Whilst no single intervention is likely to improve rates of AT on its own, a better understanding of the different economic, physical, environmental, cultural and legal circumstances that might result in more AT and less private vehicle travel is required. Given that a fuel excise intervention is able to be implemented at scale and relatively easily embedded, the potential for positive health benefits should be better explored through more comprehensive research into effects on AT behaviours [2]. This is especially the case given that relatively modest population shifts to AT based on cross-price elasticities of demand for public transport with respect to fuel price as we have modelled here may result in significant obesity and PA-related benefits.

More evidence is also required on the potential sustainability of effect of specific interventions, given that individuals may alter their travel behaviours in the longer run. These longer term changes in behaviour may result in more AT – for instance, consumers may choose to move closer to their place of work so that they can walk or cycle instead of drive if fuel taxation rises. Or it may result in less travel or less AT specifically – for instance, consumers may purchase more fuel efficient or electric vehicles or change their travel patterns altogether over time if fuel taxes increase. If however consumers become desensitised to price increases over time, the intervention may also have limited effect on travel behaviours in the longer term. Given the lack of evidence of effect, our modelling assumed effect stability over the working life (18 to 64 years). This is both a limitation and a strength of our study; limiting in the sense that this assumption may overstate the stability of effect during a person’s working years but a strength in that we make no assumption that a long-term change in travel behaviour leads to a continuation of AT behaviour upon retirement.

Our results modelled potential health impacts on commuting trips, however this is also a small proportion of total trips made by motor vehicles (and potentially affected by the intervention). Travel to and from work made up only approximately one quarter of total passenger vehicle kilometres travelled in Australia in 2014 [88]. Comprehensive data on transport behaviours is relatively limited at the national level in Australia. Our results therefore potentially underestimate the health-related benefits of an increase in fuel taxation by not taking into account travel for any other purpose. Our results also do not accurately reflect the potential impact of a fuel excise intervention on those not in the workforce, such as retirees, the unemployed or children.

The efficiency of the fuel excise tax as a source of Australian government revenue currently focuses on the desirability of tax neutrality – that is, that the tax minimises distortions to consumption decisions as much as possible. The motivation for increasing fuel excise taxation to improve rates of AT would be to purposefully distort consumer choices, and so it is likely that any such intervention would be both politically and socially sensitive (Table 7). Fuel purchases make up approximately 2.97% of the average household weekly expenditure on goods and services in Australia, with middle-income households spending slightly more (approximately 3.4% for households in the second income quintile and 3.3% for households in the third income quintile) than the lowest or highest income households (2.93% and 2.49% respectively) (Appendix 6) [89]. The intervention would therefore result in greater financial impact on middle and low-income households than high-income households (Appendix 6) and households whose main source of income is from government payments would also be relatively worse off than high-income households (Appendix 6) [89]. Future scope exists for a more detailed exploration of intervention outcomes, costs and consequences by socioeconomic groups.

**Table 7:** Second stage filter analysis of a fuel excise taxation intervention

Filter	Summary	Decision points
Level of evidence	Quantity and quality of evidence supporting association between fuel price or taxation and AT is limited. May be effective: No Level I or II evidence Modelling based on hypothetical scenario analysis	Weak evidence of effectiveness
Equity	Equity concerns: Disproportionate effect across low, middle and high-income households. Middle-income households most affected as a proportion of overall weekly household expenditure. High-income households least affected as proportion of overall weekly expenditure. Evidence suggests that public transport is less accessible for persons with disabilities, the elderly, those living in areas not well-serviced by comprehensive networks and those from disadvantaged backgrounds.	Moderate issue
Acceptability	Would require measures to be put into place to increase acceptability (for instance, revenue reinvestment to deal with potential regressivity and to ensure comprehensive public transport accessibility).	Moderate issue
Feasibility	The intervention is feasible. The feasibility of modal switch to public transport as a result of the intervention may be limited in rural areas or areas not currently well-serviced by comprehensive public transport networks. A recent Australian survey found that 30% of respondents did not use public transport to work or full-time study due to the fact that no service was available at all, with 5.5% of respondents reporting that services were located too far from home [109].	Not a major issue
Sustainability	The sustainability of effect is relatively unknown. Consumers may adjust behaviour to price rises over the longer term.	Weak evidence of sustainability
Side-effects	*Positive:* Potential for less traffic, pollution, safer environments for pedestrians and cyclists *Negative:* Potential strain on public transport networks	Significant wider positive side-effects
Policy considerations: The intervention demonstrates potential for cost-effectiveness, but is limited in terms of quality of evidence of effect and sustainability. Concerns around equity and acceptability would need to be addressed.		

Due to this potential regressivity, it is clear that social offsets would be required in order to achieve political and social acceptability. The approximately AUD1.7B in revenue that the Australian Government would stand to collect on an annual basis through the increase (AUD1.5B in additional excise based on assumed intervention effect and passenger vehicle petrol consumption in 2010 [90] and AUD154M in additional GST) could be directed towards minimising regressivity and ensuring that the other factors necessary to support a switch to AT, such as PT accessibility, are available. Convenient, low-cost, affordable and good quality PT networks would act as both enablers and motivators of a move away from the current dependence on private motor vehicle travel towards more active forms of transport [91].

Whilst there have been calls for a more central role for public health in the transport planning and policy agenda [92] our findings highlight the fact that more research into potential health impacts is required in order for public health considerations to be more comprehensively considered. Whilst the body of evidence for the environmental, social and health impacts of transport planning practices has grown in recent years, there is still a significant gap in knowledge in terms of which specific interventions may provide better social, health, environmental and economic outcomes. Parallel literature has examined the potential effectiveness and cost-effectiveness of taxing other unhealthy behaviours such as drinking alcohol or sugar-sweetened beverages [93–95] and cigarettes [96, 97]. Our review demonstrates that there may be significant obesity and PA-related benefits in using fuel excise taxation as more than a neutral revenue-raising stream. Our results however highlight the need for better knowledge on the wide range of policy levers that may encourage more physically active societies.

## Conclusions

Relatively limited evidence exists on the impacts of fuel price or taxation on obesity or PA-related behaviours. Exploratory modelling, using plausible estimates associated with modal switch to PT demonstrates that a fuel excise taxation intervention may provide small individual level benefits in a relatively small subset of the Australian population. If the effect is maintained over time however, these relatively small changes could lead to relatively large population level health gains. In order to be politically and socially favourable, a fuel excise intervention designed to increase rates of AT would however have to overcome significant equity and acceptability challenges. This could possibly occur through reinvestment of taxation revenues into initiatives such as better provision of alternative modes of transport. A range of intervention approaches is likely required to improve rates of AT, especially in countries with low prevalence. Implementation of such interventions is often incremental, and our paper provides valuable evidence on potential physical activity related health gains from a fiscal policy to make AT more appealing and driving less appealing to Australian drivers.

## Appendix 1

Search strategies.

Tables provide a summary of search strategies for the scoping review.

To be considered for inclusion studies needed to:

1. Be written in the English language in any year;
2. Be published as an academic paper in a peer review journal;
3. Be a primary study, not a review;
4. Report on (i) an obesity-related effect, or (ii) a PA-related effect of fuel taxation or price. Obesity-related effect was defined as a change in an adiposity related outcome such as weight, waist circumference or BMI. PA-related effect was defined as a change in leisure or utilitarian PA, walking or cycling.

Studies reporting on associations between fuel taxation or price and a variable representing the demand for motor vehicles (for example, motor vehicle ownership or distance travelled) were excluded because associations do not necessarily reflect a shift to more AT, but may better reflect a change in discretionary or other travel behaviours (for example, trip purpose or timing). The reference lists of included studies were also searched.

**Table 8:** Scoping review of published associations between obesity, PA, walking or cycling and fuel price or taxation search strategy

Database	Intervention terms	Outcome terms
EBSCOHost All databases, peer-reviewed only	Petrol* pric* OR petrol tax* OR gasoline pric* OR gasoline tax* OR fuel pric* OR fuel tax*	Physical activity OR “active transport*” OR bicycl* OR walk* OR pedestrian OR Obesity OR weight gain OR BMI OR “body mass index” OR “energy balance” OR “energy expenditure”
Web of Science		
*Conducted May 2016*		

**Table 9:** Scoping review of published cross price elasticities of public transport demand

Databases	Combination of search terms used
EBSCOHost (All databases, peer-reviewed only) Web of Science	Public transport*, transit, meta-analysis, review, systematic review, elasticit*
GoogleScholar First 20 pages searched (10 results per page)	Cross price elasticity and public transport and Australia
*Conducted May 2016*	

## Appendix 2

Tables provide details on the strength of evidence assessment.

**Table 10:** Strength of evidence assessment using STROBE statement, scoping review studies

Quality criteria		Specification of scores	Score
1	Study type	Cross-sectional	0
		Longitudinal	1
2	Exposure/s	Not clearly reported, no data sources given	0
		Clearly reported, with data sources given	1
3	Outcome	Self-reported	0
		Objectively measured (at least one timepoint where applicable)	1
4	Sample size	Small (*n* < 500) or not explicitly reported	0
		500–10,000	1
		>10,000	2
5	Completeness of data	Data available for <80% of participants or not reported	0
		Data available for ≥80% of participants	1
6	Statistical methods	Not clearly reported	0
		Clearly reported	1
7	Confounding	Not controlled for confounders	0
		Attempted to control for confounders	1
8	Descriptive data	Not clearly reported	0
		Clearly reported	1
9	Clear presentation of results of associations of interest	No table listing results and significance	0
		Table listing results and significance	1
*Total (highest possible)*			*10*

## Appendix 3

Table provides data parameters for the intervention effect estimate.

**Table 11:** Intervention effect, modeled to physical activity and BMI effect, main scenario

Age (years)	18	19	20–24	25–29	30–34	35–39	40–44	45–49	50–54	55–59	60–64
*Males*											
Time spent to access PT per week (mins)	90.19	90.19	90.19	90.19	86.17	86.17	85.94	85.94	90.19	90.19	92.45
METs to kcal/min	3.21	3.38	3.45	3.67	3.67	3.67	3.92	3.81	3.84	3.84	3.83
KJ per week from intervention	289.76	304.69	311.38	330.81	316.26	316.26	337.13	327.67	346.47	346.67	354.21
Weight effect (kg)	−1.73	−1.82	−1.86	−1.98	−1.89	−1.89	−2.01	−1.96	−2.07	−2.07	−2.12
BMI effect (kg/m^2^)	−0.55	−0.56	−0.59	−0.63	−0.60	−0.59	−0.64	−0.63	−0.67	−0.68	-0.70
*FEMALES*											
Time spent to access PT per week (mins)	89.29	89.29	89.29	89.29	88.79	88.79	90.32	90.32	90.06	90.06	96.94
METs to kcal/min	2.67	2.79	2.96	3.01	3.05	3.10	3.18	3.26	3.13	3.18	3.24
KJ per week from intervention	238.46	249.49	264.03	268.69	270.69	275.82	287.37	294.32	282.29	286.07	314.51
Weight effect (kg)	−1.42	−1.49	−1.58	−1.61	−1.62	−1.65	−1.72	−1.76	−1.69	−1.71	−1.88
BMI effect (kg/m^2^)	−0.52	−0.53	−0.58	−0.59	−0.60	−0.61	−0.65	−0.67	−0.64	−0.66	−0.74

Table notes *PT* public transport, *Mins* minutes, *METs* metabolic equivalent task, *kcal/min* kilocalories per minute, *KJ* kilojoules, *kg* kilograms. All values rounded to 2 decimal places

## Appendix 4

Parameters for uncertainty analysis.

Tables provide summaries of one-way and “plausible case” sensitivity analyses input parameters.

**Table 12:** One-way sensitivity analysis parameters

Parameter	Value used in primary analysis	Value/s used in one-way sensitivity analyses	Source/s
*Intervention effect*			
Cross price elasticity for public transport with respect to fuel price	0.07	0.82, 1.15	Sensitivity analysis values sampled from a normal distribution, as reported by Holmgren 2007 [78]. Derived increase in the prevalence of PT commuting of 7.2% and 10.1% respectively.
Average distance a person will walk to access public transport (metres)	400 m	800 m	Based on ‘rule of thumb’ planning guidelines

**Table 13:** “Plausible case” scenario for sensitivity analysis

Parameters for ‘plausible case’ scenario analysis	Mean values and 95% UI^a^ (where applicable)		Sources and assumptions
*Intervention effect*			
Cross price elasticity for public transport with respect to fuel price	0.37 (95% UI -0.24-0.97)		Sampled from a normal distribution, taken from mean cross price elasticity as reported by Holmgren 2007 [78]. Derived increase in the prevalence of PT commuting of 3.3% [38]. Modelled to PA/BMI effect. Assumed all new public transport users were previous car drivers, a reasonable assumption given the high prevalence of driving to work in Australia [38].
Average annual retail fuel price (national, metropolitan) (cents per litre)	125.39 (95% UI 124.95–125.83)		Sampled from a gamma distribution, from national metropolitan fuel price [102]. As per primary analysis.
Prevalence of using public transport for commuting purposes	*Males* 18y-4.5% 19y-5.8% 20-24y-8.5% 25-29y-11.7% 30-34y-11.1% 35-39y-9.1% 40-44y-7.4% 45-49y-6.3% 50-54y-5.8% 55-59y-4.9% 60-64y-3.3%	*Females* 18y-6.9% 19y-8% 20-24y-11.1% 25-29y-13.1% 30-34y-9.9% 35-39y-6.8% 40-44y-5.9% 45-49y-5.7% 50-54y-5.3% 55-59y-4.5% 60-64y-2.9%	ABS Census 2011 [38]. As per primary analysis.
Marginal MET value for walking to access public transport	3		MET value for walking to work or class of 4 from Ainsworth et al. 2011 [81], adjusted for inactivity. Sampled using a lognormal distribution (stdev 1.6 from Gotschi et al. 2015 [103]).
Average distance a person will walk to access public transport (metres)	800		Based on ‘rule of thumb’ planning guideline for distance walked to bus/tram access points.
Comfortable gait speed (cm/s)	As per primary analysis.		
Number of weeks of intervention effect (averaged over year)	As per primary analysis		

## Appendix 5

Table provides summary of results of quality assessment of included studies in scoping review.

**Table 14:** Results of quality assessment of included studies in scoping review

Study	Quality assessment criteria									QA score
	1	2	3	4	5	6	7	8	9	
BMI										
Courtemanche 2011 [57]	0	1	0	2	1	1	1	1	1	8
Rabin et al. 2007 [58]	0	1	0	0	1	1	0	1	1	5
Sun et al. 2015 [59]	0	1	0	0	1	1	0	1	1	5
Mean (BMI studies)										6
Physical activity										
Hou et al. 2011 [60]	1	1	0	1	0	1	1	1	1	7
Sen 2012 [61]	0	1	0	2	1	1	1	1	1	8
Sen et al. 2014 [62]	0	1	0	2	0	1	1	1	1	7
Mean (PA studies)										7.3
Cycling										
Buehler & Pucher 2012 [63]	0	1	0	0	0	1	1	1	1	5
Dill & Carr 2003 [68]	0	1	0	0	1	0	1	1	0^a^	4
Pucher & Buehler 2006 [67]	0	1	0	0	1	1	1	1	1	6
Rashad 2009 [64]	0	1	0	2	0	1	1	1	1	7
Smith & Kauermann 2011 [65]	0	1	1	0	1	1	1	1	1	7
Mean (cycling studies)										5.8
Walking										
Ryley 2008 [66]	0	0	0	1	0	1	1	0	1	4
*Mean QA for all study inclusions*										6

^a^Study reports selected findings, but not for relevant variables here

## Appendix 6

Table provides summary of potential equity effects of the proposed intervention.

**Table 15:** Potential equity implications of the intervention

Parameter	All households (mean)	Gross household income quintile					Source
		Lowest	Second	Third	Fourth	Highest	
Average weekly household expenditure on fuel 2009–10 (AUD)	36.66	16.36	27.6	38.55	47.00	53.87	ABS Household Expenditure Survey [89] and average annual retail petrol price per litre [102]
Average total weekly household expenditure, all goods and services 2009–10 (AUD)	1236.28	559.04	814.94	1169.47	1479.45	2159.74	
Proportion of average weekly household expenditure spent on fuel (pre-intervention)	2.97%	2.93%	3.39%	3.30%	3.18%	2.49%	
Proportion of average weekly household expenditure spent on fuel (incorporating price rise of AUD0.10 per litre)	3.23%	3.18%	3.68%	3.59%	3.46%	2.71%	
Change in proportion of weekly household expenditure on goods and services spent on fuel as a result of the intervention	0.26%	0.26%	0.30%	0.29%	0.28%	0.22%	
Parameter	Main source of household income						Source
	Aged pension	Income disability and carer payments	Unemployment and study payments		Family support payments	Government pensions and allowances	
Average weekly household expenditure on fuel 2009–10 (AUD)	18.24	24.50	28.72		29.50	20.31	ABS Household Expenditure Survey [89] and average annual retail petrol price per litre [102]
Average total weekly household expenditure, all goods and services 2009–10 (AUD)	564.82	726.94	713.14		834.09	612.94	
Proportion of average weekly household expenditure spent on fuel (pre-intervention)	3.23%	3.37%	4.03%		3.54%	3.31%	
Proportion of average weekly household expenditure spent on fuel (incorporating price rise of AUD0.10 per litre)	3.51%	3.67%	4.38%		3.85%	3.60%	

## Abbreviations

ABS: Australian Bureau of Statistics
AIHW: Australian Institute of Health and Welfare
AT: Active transport
AUD: Australian dollar
B: Billion
BITRE: Bureau of Infrastructure
BMI: Body mass index
cm: Centimetre
CVD: Cardiovascular disease
DALY: Disability adjusted life year
EU: Energy units
GBD: Global Burden of Disease
GST: Goods and services tax
HALY: Health adjusted life year
ICER: Incremental cost-effectiveness ratio
kc: Kilocalories
kg: Kilogram
KJ: Kilojoules
km: Kilometre
LR: Long-run
LTPA: Leisure time physical activity
M: Million
ME: Marginal effect
MET: Metabolic equivalent task
mins: Minutes
OECD: Organisation for Economic Co-operation and Development
OLS: Ordinary least squares
PA: Physical activity
pp.: Per person
PRISMA: Preferred Reporting Items for Systematic Reviews and Meta-Analyses
PT: Public transport
QA: Quality assessment
RIS: Regulatory impact statement
s.e.: Standard error
sec: Second
SR: Short-run
st.dev.: Standard deviation
STROBE: Strengthening the Reporting of Observational Studies in Epidemiology Transport and Regional Economics
UI: Uncertainty interval
VISTA: Victorian Integrated Survey of Travel and Activity
VKT: Vehicle kilometres travelled
VMT: Vehicle miles travelled
VOC: Vehicle operating cost
